# Patient experience of scar assessment and the use of scar assessment tools during burns rehabilitation: a qualitative study

**DOI:** 10.1093/burnst/tkab005

**Published:** 2021-06-01

**Authors:** Kate Price, Naiem Moiemen, Laura Nice, Jonathan Mathers

**Affiliations:** 1 College of Medical and Dental Sciences, University of Birmingham, Birmingham, UK; 2 Scar Free Foundation Centre for Conflict Wound Research, Queen Elizabeth Hospital, University Hospitals Birmingham NHS Foundation Trust, Birmingham, UK; 3 NIHR Surgical Reconstruction and Microbiology Research Centre, Queen Elizabeth Hospital, Birmingham, UK; 4 Institute of Applied Health Research, University of Birmingham, Birmingham, UK

**Keywords:** Burns, Scarring, Scar assessment, Scar assessment scales, Qualitative research, Interviews, Objective scar assessment

## Abstract

**Background:**

Scar assessment plays a key role during burns aftercare, to monitor scar remodelling and patients’ psychosocial well-being. To aid assessment, subjective scar assessment scales are available that use health-care professionals’ and patients’ opinions to score scar characteristics. The subjective scales are more widely used in clinical practice over objective scar measures. To date, there is no research that considers patients’ views on scar assessment and the role of subjective and objective assessment tools. Therefore, the aim of this qualitative study was to explore patients’ perspectives on scar assessment and the utility of scar assessment tools during burns rehabilitation.

**Methods:**

Semi-structured interviews were conducted with 10 adult burn patients who were being reviewed in clinic for scarring. Participants were recruited via their clinical care team and research nurses at the Queen Elizabeth Hospital, Birmingham, UK. Topics covered during interview included patient experience of scar assessment, the use of scar assessment tools and discussion surrounding important factors to be addressed when assessing scars. A thematic analysis using the Framework Method was conducted.

**Results:**

Participants identified key subthemes that contribute towards the overarching theme of patient-centred scar assessment. These are: patient-led care; continuity in care; learning how to self-manage scarring; and psychological assessment. Links were demonstrated between these subthemes and the remaining themes that describe scar assessment strategies, indicating their potential patient-centred contributions. The subjective opinions of clinicians were found to be valued above the use of subjective or objective scar assessment tools. Scar assessment scales were perceived to be a beneficial method for self-reflection in relation to psychosocial functioning. However, minimal feedback and review of completed assessment scales led to uncertainty regarding their purpose. Patients perceived objective tools to be of primary use for health-care professionals, though the measures may aid patients’ understanding of scar properties.

**Conclusions:**

Scar assessment tools should be used to support, rather than replace, health-care professionals’ subjective judgements of scarring. Adapting the way in which clinicians introduce and use scar assessment tools, according to patient needs, can support a patient-centred approach to scar assessment.

Highlights

- Key subthemes relating to scar assessment contribute towards patient-centred care.
- Subjective scar assessment scales can provide patients with an opportunity for psychological reflection.
- Objective scar assessment tools may support patients’ understanding of physical scar properties.
- Scar assessment tools should support not replace clinicians’ professional judgements on scarring.
- Adapting the way in which clinicians introduce and use scar assessment tools, according to patient needs, can support a patient-centred approach to scar assessment.

## Background

Burns injuries and the associated scarring can cause lifelong disfigurement, loss of function and psychosocial implications [1]. Physical scar complications are common, including hypertrophic changes and contractures that can restrict movement [2–5]. Previous research has demonstrated links between the presence of scarring, emotional distress and the development of mental health conditions [6–7]. Therefore, assessment and treatment of scarring has the potential to reduce impact upon health-related quality of life (HRQoL) [8, 9].

Scar assessment is crucial to determine scar severity, monitor progression and aid clinical decision making [5, 10]. It can also provide insight into the sensory, physical and emotional consequences of scarring from a patient’s perspective [11]. Assessment can be conducted in a subjective manner, through the use of scar assessment scales and clinicians’ judgements of scarring, or objectively, utilizing technology to produce quantitative scar measurements [10, 12]. To assess scar properties alone, the Patient and Observer Scar Rating Scale (POSAS) and the Vancouver Scar Scale (VSS) are favoured in clinical practice, due to their low cost and user-friendly nature [3, 12]. A systematic review by Tyack et al. deemed the POSAS to be superior to the VSS and 16 other assessment scales [13]. However, due to indeterminate clinimetric quality ratings across multiple measures, including validity for the POSAS, a single ‘gold standard’ scar assessment scale has not been determined.

The Brisbane Burn Scar Impact Profile (BBSIP) was designed to assess HRQoL [14]. It focuses upon patient-specific scar symptomology and components of quality of life, for example, daily activities, social interactions and emotional well-being [11]. Studies have demonstrated validity for multiple psychometric measures covered within the tool; however, patients’ views regarding the value of the BBSIP have not been explored since its implementation in clinical practice [11, 15].

Previous research has demonstrated the inter-user variability of scar assessment scales, with factors such as clinician experience and patient psychological distress influencing scoring [16]. Objective measures can potentially be used to overcome this limitation, for example, high-frequency ultrasound scanners and cutometers are able to distinguish between hypertrophic and non-hypertrophic scarring [17]. However, they do not address HRQoL associated with scarring, such as tools like the BBSIP. Furthermore, the expense of introducing technology has limited use outside the research context [18].

Current evidence has demonstrated the rationale for using scar assessment tools and has evaluated the validity of existing measures [10, 18–20]. However, to date there has not been any research that has explored patients’ views on scar assessment and the utility of assessment tools. Exploring the patient perspective can help to establish whether scar assessment is conducted in a beneficial manner and determine the patient relevance of assessment tools. Examining patients’ experiences could prove valuable, given that researchers have been unable to identify a superior assessment method and with the emergence of data supporting the use of objective scar measures [13, 17, 21].

Previous qualitative research has demonstrated that important outcomes for patients throughout scar management are often complex and encompass more than just scar characteristics [22]. There is also no clear association between scar severity and psychological morbidity following burn injury [23]. It is important to consider the interplay between scores recorded through assessment tools and patients’ views of their scarring, to determine whether the measurements translate into a meaningful value for patient use.

This qualitative study was designed to allow incorporation of patients’ views into the evidence base surrounding scar assessment and facilitate holistic decision making regarding the use of assessment tools. The aim was to explore patients’ views and experiences of scar assessment and the use of both subjective and objective scar assessment tools in an outpatient burns rehabilitation setting.

## Methods

Methodology is reported according to the Consolidated Criteria for Reporting Qualitative Research (COREQ) checklist [24].

### Study design

This was an exploratory qualitative research study focused on adult burns patients’ experiences of scar assessment and the use of scar assessment tools during burns rehabilitation.

### Ethics

This research was approved by the East of England – Cambridge East Research Ethics Committee (19/EE/0359).

### Patient involvement

Adult patients with experience of a burn injury were involved in the study design. A patient and public involvement (PPI) meeting with an established group of burns patients who regularly contribute to the design of trauma-related research at the Queen Elizabeth Hospital, Birmingham, UK, ensured that the study aims and methods were patient-centred. The interview topic guide (Supplementary file 1) was discussed in detail. A second virtual PPI meeting was conducted to discuss the results of this study.

### Sampling and recruitment

Adult patients were recruited from the Queen Elizabeth Hospital, Birmingham, UK, a tertiary centre for burn injuries, where scar assessment is conducted using the BBSIP and both the patient- and observer-reported versions of the POSAS. The site has also been used to trial objective scar assessment tools. At the site, scar assessment scales are usually completed during outpatient appointments with therapists. The scales are completed on assessment following initial wound healing and are updated if there is a significant change to treatment, such as before and after reconstructive surgery or if there is a noticeable deterioration or improvement in scarring. The scales are routinely updated at 12 months or at the point of discharge from the outpatient clinic if this is sooner. A purposive sample of 10 participants was identified, 7 by occupational therapists during outpatient clinics and three by research nurses during a PPI meeting held on site. Two other patients were approached but did not wish to take part in an interview due to personal circumstances. Patients were included if they were over the age of 18 and receiving scar treatment or being reviewed by a clinician for scarring caused by burn injury. Participants had to be able to understand and communicate in English to take part in the study. We attempted to achieve diversity within the sample according to total body surface area of burn injury, sex and age. Potential participants were provided with an information sheet and completed a contact detail form, giving consent to be contacted by a researcher (KP). After a minimum of 48 hours the researcher contacted potential participants to provide further information about the study, answer questions and to arrange an interview. Further recruitment to the study was curtailed due to the coronavirus pandemic as outpatient appointments at the recruiting site were cancelled. However, data saturation of key themes had already been achieved at this point.

### Data collection

Semi-structured interviews were conducted to enable rich data to be obtained for analysis and were deemed appropriate to allow exploration of potentially sensitive topics [25]. The interviews were conducted at the University of Birmingham (n = 1), patients’ homes (n = 3) or over the telephone (n = 6), according to participant preference. Interviews were conducted by KP, a female medical student independent of the patients’ clinical team, with training and support being provided by JM, an experienced qualitative researcher. Interviews lasted between 18 and 70 minutes. Verbal or written informed consent was obtained prior to interview and a participant background questionnaire was completed, allowing socio-demographic and burn characteristics to be monitored. An interview topic guide (Supplementary file 1) was designed according to the research aims, feedback from the PPI group and existing relevant literature. Interview conduct was developed iteratively allowing emerging insights to influence further data collection [26]. Interviews were patient-centred, and participants were enabled to guide discussion naturally. Topics explored during the interview included: participants’ experiences of acquiring a burn injury and undergoing rehabilitation; patient accounts and expectations relating to scar assessment and their views on the use of subjective and objective scar assessment tools. Field notes were collected in line with guidance by Phillippi and Lauderdale, to aid contextualization and understanding of data [27].

### Data analysis

Interviews were audio recorded prior to transcription clean verbatim. A thematic analysis proceeded using audio-recordings, transcriptions and field notes, guided by the Framework analytical approach [28]. Initial data immersion involved familiarization through re-listening to interviews, reading transcripts and reviewing field notes. Open coding of data was then undertaken in NVivo version 12, with iterative development of an analytic framework [29]. This coding framework was applied to each interview (indexing) and a matrix created to facilitate associative analysis and to further data interpretation. A sample of 10 interviews were coded by KP, three transcripts were also coded by JM. Initial coding and categorizations were refined via this process. Thematic interpretations were discussed amongst the research team and with other experienced qualitative researchers to aid thematic development. KP took a reflexive stance throughout, considering how close involvement in data collection and medical training had the potential to influence interpretation. Analysis of later interviews provided no additional conceptual insights, therefore it was judged that data saturation had been attained concerning major analytic themes [30].

## Results

### Sample characteristics

The sample consisted of two male and eight female participants from across the Midlands region of the UK, representing a range of ages (18–70) (Table 1). Three participants were from a non-white background. All participants were being reviewed in clinic for scarring from burn injuries (acquired between less than 1 year and over 40 years previously). Total body surface area of burn injury ranged from 1% to 68%, with the majority flame-injured. All participants had experience of completing subjective scar assessment scales and two participants had first-hand experience of objective scar assessment measures.

**Table 1:** Patient demographics and burn injury characteristics

**Participant identifier**	**Gender**	**Age (years)**	**Ethnic group**	**Marital status**	**Highest educational achievement**	**Employment status**	**Date of burn injury (years/months since injury)**	**Type of burn injury**	**TBSA (%)**	**Skin grafts performed**
1	Female	18–25	White	Single	GCSEs	Employed	09/2001 (18 yr)	Scald	<10	Yes
2	Female	31–40	Asian	Married	No formal qualifications	Housewife	05/2019 (11 m)	Flame	<10	No
3	Female	31–40	African Caribbean	Unassigned	GCSEs	Employed	10/2019 (6 m)	Scald	<10	Yes
4	Male	25–30	White	Single	No formal qualifications	Employed	07/2019 (9 m)	Flame	10–20	No
5	Female	41–50	White	Cohabiting	A levels	Unemployed	02/2019 (1 yr)	Chemical	10–20	Yes
6	Female	41–50	Asian	Married	A levels	Employed	01/1974 (46 yr)	Flame	21–30	Yes
7	Female	31–40	White	Single	Undergraduate degree	Self-employed	04/2017 (3 yr)	Flame	31–40	Yes
8	Female	41–50	White	Single	No formal qualifications	Unemployed	04/2018 (2 yr)	Flame	61–70	Yes
9	Female	61–70	White	Widowed	Higher degree	Retired	10/1957 (62 yr)	Flame	61–70	Yes
10	Male	61–70	White	Cohabiting	GCSEs	Unemployed	10/1993 (26 yr)	Flame	61–70	Yes

*TBSA* total bosy surface area, *GCSEs* general certificate of secondary education

### Themes

Four themes provide insight into patients’ experiences of scar assessment and scar assessment tools:

- Patient-centred scar assessment
- The utility of scar assessment scales
- The value of subjectivity
- The potential role of objective scar measures.

The theme ‘*patient-centred scar assessment*’ is an overarching theme. Its subthemes (*patient-led care*, *continuity in care*, *learning how to self-manage scarring*, *psychological assessment*) describe the elements of clinic that contribute towards conducting scar assessment in a patient-centred manner.

**Figure 1.**
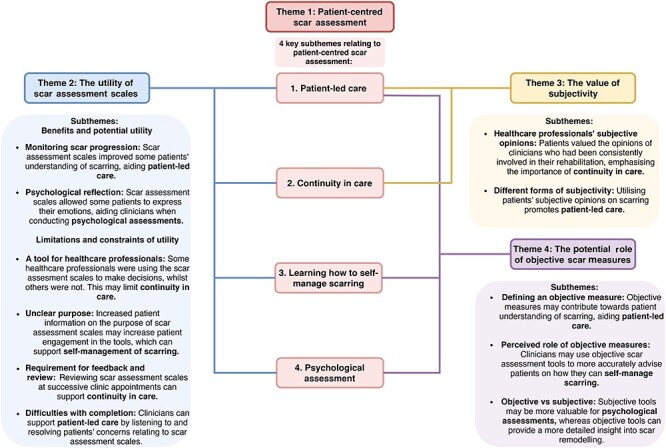
Diagram illustrating links between the four key subthemes of patient-centred scar assessment and other themes which describe scar assessment strategies

The remaining three themes describe strategies used to assess scars and their links to the key components described by the subthemes of ‘*patient-centred scar assessment*’. Figure 1 illustrates and provides examples of this.

### Patient-centred scar assessment

Participants indicated that attending clinic for scar assessment is a central part of their rehabilitation. This relates to four key subthemes, described by participants, which have positively influenced their experience of clinic. Many expressed that these components enable scar assessment and management to be tailored towards their individual needs. This infers that they are essential to achieve a patient-centred approach to scar assessment.

#### Patient-led care

Many participants felt that they were included as a member of the clinical team, allowing them to be involved in guiding scar assessment. For example, one participant discussed how clinicians might direct questioning to tailor scar assessment towards patients’ rehabilitation goals:

*‘The physio girls will go, “How’s your fingers?” And I will go, “I wish I could have it straight so I could grip more”. So we talk about that.’—*Interviewee 8.

Participants described the relationships formed with health-care professionals and how this complemented patient-led care. Participants explained that they could contact health-care professionals outside of clinic, meaning they could inform them of emerging issues relating to their scarring:

*‘The physio was really good, she became my coordinator and point of contact and she was only a phone call away if you needed anything.’—*Interviewee 8.

There are indications in the data that over time scar assessment becomes more patient-led as patients gain experiential knowledge about scarring. Their involvement in clinical discussions was described by Interviewee 10, who was 26 years post-burn injury:

*‘I’ll do all the telling them what I want and what I have been doing and where it’s been working … they will just suggest well carry on doing that or don’t use this … and then we come to an agreement.’—*Interviewee 10.

Loss of independence was highlighted as a negative consequence of burn injury. Being involved in scar rehabilitation may provide patients with a regained sense of control:

*‘It gives me time to plan ahead and to think about … what I want to do or what I might not want to do.’—*Interviewee 5.

Participants stressed that assessing the impact of scarring on daily activities is a necessity during clinic. Patient-led care can support this by allowing patients to express their priorities for scar rehabilitation, which can aid in improving quality of life. Participants explained the impact of scarring on daily life:

*‘Movement, strength, being able to grip things like to open up a can, I couldn’t do that at all, lifting pans I had to have help with. Just normal day-to-day stuff you take for granted.’—*Interviewee 5.

Some interviewees identified instances where they felt that secondary issues relating to scarring were not adequately assessed by clinicians. For example, Interviewee 6 talked about a lack of focus on loss of sensation due to scarring, suggesting that the expectation of patient-led care had not been met on this occasion:

*‘There was no mention or talk about that, the feeling, the sensation in my back … I would have thought comes under scarring … and I’m thinking well I still can’t feel anything in my back, how long is it going to take?’—*Interviewee 6.

#### Continuity in care

Burn injuries can require significant hospitalization and rehabilitation, as described by participants during interview. This provides opportunity for patients to become well acquainted with their care team. This familiarity between patients and their clinical team provided some participants with psychological reassurance:

*‘I was really lucky with my physio … you see her so often that you build confidence and trust … if something wasn’t right she was an expert so she would go and get you the help that you needed. So I think she reassured me a lot, she became a big strength for me.’—*Interviewee 8.

Being seen by the same health-care professionals meant that participants did not have to repeatedly disclose traumatic details of how they acquired their burn. This had associated emotional benefits:

*‘It’s not like I have to tell the person, because it’s tiring to keep telling the same thing, repeating myself. Good thing it’s the same people.’—*Interviewee 4.

Continuity in care comprises more than attending appointments with the same clinicians. Having consistency and agreement amongst members of the clinical team was also emphasized. On occasion, participants noted a lack of continuity between members of their clinical team, leading to inconsistent advice:

*‘I was getting a lot of conflicting information, some people were saying you might be lucky, you might have escaped needing skin grafts and others were like no you need skin grafts. So I was exceptionally concerned.’—*Interviewee 7.

The value of continuity was more apparent for authoritarian members of the clinical team, such as consultants. This related to the perceived competence of health-care professionals.

#### Learning how to self-manage scarring

During clinic, participants valued the opportunity to learn about scar self-assessment and management techniques. Interviewees felt that this allowed them to be more actively involved in care:

*‘They always made sure that I was aware* [how] *to massage the scars, moisturising them is the most important thing you can do.’—*Interviewee 8.

Self-management was positively encouraged by clinicians:

*‘I have kept up with all the exercises and the last time I saw the physio she was really amazed with the progress and she said, “You’ve managed to rehabilitate yourself, you’ve been a model patient”.’—*Interviewee 5.

Many of the scar management techniques described by participants focus on regaining movement, complementing patients’ ability to carry out daily activities:

*‘The physios if they think I need any stretching or exercising they will tell me what to do and I can do that.’—*Interviewee 10.

Movement was also described as a crucial element of scar assessment within the subtheme *patient-led care*.

#### Psychological assessment

Participants highlighted the negative impact that scarring can have on psychological well-being. Many indicated that this had been informally addressed in clinic, inferring that clinicians were conducting assessments of patients’ emotional health:

*‘They are keeping an eye on you psychologically in case there’s any referral needed for any extra support.’—*Interviewee 7.

One participant suggested that having an established rapport with clinicians allowed her to feel comfortable talking about her emotional well-being, reiterating the value of *continuity in care*:

*‘They chat to you like friends … they reassure you that it’s alright to feel like that.’—*Interviewee 8.

There was variation amongst participants, depending on when they acquired their burn injury, regarding the extent to which the psychological impact of scarring had been addressed in clinic. Patients with more recent burn injuries felt that there had been less discussion regarding the psychological elements of their scarring, whereas patients who were further progressed in their burns rehabilitation indicated that this had been adequately addressed.

For example, one participant with a more recent injury explained that she did not receive psychological support, inferring that it would have been beneficial for her recovery:

*‘These accidents there is a psychological factor as well. If somebody is doing sympathy with you, putting a hand on your head, is making you calm sometimes, that helps a lot. I would definitely say I did not receive anything from anyone.’—*Interviewee 2.

The significance of assessing the psychological impact of scarring during the early stages of burns rehabilitation was reinforced by Interviewee 7:

*‘There could be more psychological intervention from the psychiatrists and counsellors.’—*Interviewee 7.

This contrasted with participants who had greater experience of living with scarring, who indicated that the informal assessments were sufficient:

*‘As long as they take an interest in the emotional side of things, because it is a huge part of burns.’—*Interviewee 5.

### The utility of scar assessment scales

#### Benefits and potential utility

The majority of participants were aware that scar assessment scales had been used during clinic and were able to comment upon useful aspects of the scales.

##### Monitoring scar progression

When questioned about the personal use of scar assessment scales, some participants suggested that they can help to track scar progression. For example, one interviewee noted that the numerical scores achieved on scales such as the VSS and POSAS can be used to monitor scar properties:

*‘I remember what I had the first time when they asked me … I* [scored] *it a four but now I can say from day one up to now I can score it a six.’—*Interviewee 3.

The importance of assessing the impact of scarring on daily activities was reiterated as some participants felt that these are the most useful elements of a scar assessment scale:

*‘Making sure that I can do everyday activities was quite good, just because it allows me to write if I am not able to do that which would be quite important, because I need to be able to do everything.’—*Interviewee 1.

##### Psychological reflection

Participants referred to the psychological assessments conducted through scar assessment scales, such as the BBSIP, more frequently than the assessment of physical scar properties. It was viewed that the scales help health-care professionals to gain an insight into the impact of scarring on patients’ psychosocial well-being:

*‘The part in the tick box where it asks you how your scars and your burns affect your life on a social level … when the doctors or the nurses they look at that they can come up with a plan how they can make you more comfortable.’—*Interviewee 8.

Interviewee 8 continued to explain that scar assessment scales enabled them to articulate their feelings to their clinical team. The participant expressed that the scales allowed them to avoid feelings of guilt, relating to admission of social difficulties:

*‘When I see the surgeon or some of the other staff you can feel a little bit embarrassed to say on a social level, and I feel horrible because for me I look at what they have done for me and they saved my life and it’s just scars, and you feel like you don’t want to … but we go home and then we live with it. So sometimes it’s easier to write it down.’—*Interviewee 8.

One interviewee explained that the scales may prevent patients from having to re-explain their injuries to multiple health-care professionals, providing some psychological reassurance through continuity even if their usual clinicians are not available:

*‘Sometimes the one* [that saw] *me last time she was not available, it could help another person who cover her to know where I am … it’s not like I have to tell the person.’—*Interviewee 3.

Interviewee 4 reported personal psychological benefits from completing scar assessment scales:

*‘Putting pen to paper with it … it’s useful aiding me getting it off my chest, talking about, the more I talk about it, it makes me feel better.’—*Interviewee 4.

Interviewee 7 emphasized the requirement for psychological support during the early phases of rehabilitation.

*‘In the first 18 months the psychological impact of it, I think everybody is so focused on the healing process that maybe the psychological bit gets overlooked … If it was dealt with at an earlier stage I think people would have less problems later down the process.’—*Interviewee 7.

#### Limitations and constraints of utility

Due to their perceived purpose, participants generally felt that scar assessment scales were a less significant part of their experience in clinic and indicated areas where their use could be enhanced.

##### Unclear purpose

Some participants perceived scar assessment scales to be mandatory paperwork, highlighting that patients and clinicians may not be optimizing the scales as a reflective opportunity. In particular, participants mentioned the length of the scales and indicated that they can be viewed as a chore:

*‘They bring the questionnaire and it’s quite a few pages long.’—*Interviewee 4.

Interviewee 3 suggested that this negative perception was reinforced by some health-care professionals:

*‘They even say okay you’ve got homework to do so just fill it out.’*—Interviewee 3.

In particular, participants who had been attending outpatient clinic for longer emphasized a perceived lack of relevance regarding the scales:

*‘I’ve had to sit there and fill forms out … I don’t know how it helps me.’—*Interviewee 10.

This may relate to *patient-led care*, where more experienced patients were found to be increasingly involved in directing scar assessment.

##### A tool for health-care professionals

Many participants believed that scar assessment scales were primarily designed as an informative tool for health-care professionals, with patients experiencing secondary benefits. Participants felt that they were used to inform clinicians about their scarring:

*‘It gives* [clinicians] *a better idea of how the person is coping physically and emotionally and where you feel you could probably go forwards.’—*Interviewee 5.

Another participant noted that some health-care professionals were making decisions based on the results of the scales. However, this conflicted with decisions made by health-care professionals who were not using the scales, highlighting the importance of continuity:

*‘They were going to give an injection which will resolve lots of pain. But now they seem to be changing mind even going worse after this questionnaire.’—*Interviewee 2.

##### Requirement for feedback and review

Many participants were unaware of how scar assessment scales were used following completion, exacerbating the idea that they are designed for health-care professionals. They noted that during clinic they did not receive any feedback following completion or review of previous scores:

*‘Unless we got some follow-up at a later stage to say right you know we filled out these questionnaires, this is what’s happening with this, and therefore this is what we’re going to be looking at treating you or going forward this is what will be happening, I don’t see it going anywhere.’—*Interviewee 6.

Lack of feedback and review of scar assessment scales was frustrating and undermined patients’ willingness to complete them:

*‘I think it’s only fair that we have some feedback and you feel as though the time isn’t wasted.’—*Interviewee 6.

Within the subtheme *monitoring scar progression* some interviewees indicated that scar assessment scales can provide information about scar progression. Interviewee 5 went on to reflect on how review of the scales may contribute towards this:

*‘I could say look back at when I first transferred to* [hospital] *and go oh yeah I’ve come quite a long way since then, and some days I feel like I am just wading through treacle and I am not getting anywhere … having a questionnaire to remind you of how far you’ve progressed I think would be wonderful.’—*Interviewee 5.

#### Difficulties with completion

Some interviewees expressed difficulties with particular elements of scar assessment scales. For example, the location of scarring affected one participant’s ability to complete the scales:

*‘It was asking about two places on my back that I have never seen, and I can’t feel, I have no sensation in my back, so when it said how does your back feel, it doesn’t feel anything.’—*Interviewee 9.

Interviewee 9 explained that she experienced daily variation in problematic scar areas and therefore felt that the scales were too generic to reflect her complex scarring:

*‘It definitely needs to be more specific rather than generic, wide-ranging questions.’—*Interviewee 9.

Participants with longer experience of scarring noted that they had learnt to adapt their daily life around their scars. This was viewed positively, allowing patients to regain normality following injury. However, when completing scar assessment scales one interviewee struggled to recall the feeling of their body prior to their burn injury, causing difficulties when numerically scoring their scars.

*‘I ain’t normal so it wouldn’t be one to ten, I’m already below the zero point … you get used to having problems.’—*Interviewee 10.

### The value of subjectivity

Throughout all interviews, reference was made to the use of subjective judgements to assess scarring. This includes patients’ personal views, the opinions of health-care professionals and scar assessment scales.

#### Health-care professionals’ subjective opinions

All participants had received judgements and subsequent advice from health-care professionals relating to their scarring. Many interviewees placed value on the subjective opinions of clinicians, due to their professional experience:

*‘It’s not so much of the scarring, I think it’s the information I am getting from the doctors … because they are the professionals.’—*Interviewee 6.

Subjective advice allowed some participants to understand the rationale behind their scar therapy:

*‘They have been really informative about the whole process: “we can’t do this until this has happened”, and they have been really good in terms of saying “well this is possibly the future operations that you will need”.’—*Interviewee 5.

As illustrated within the subtheme *continuity in care*, it was found that participants received reassurance and valued the opinions of clinicians who had been consistently involved in their rehabilitation:

*‘You see the same people through from start to finish and everybody’s burn is so different that they’re assessing you, they are looking after you, and they’re comforting you, giving you reassurance.’—*Interviewee 7.

Whilst many participants were comfortable receiving clinicians’ subjective assessments on scar properties, their views on patient-specific scar symptomology were deemed inappropriate by one participant:

*‘The doctor asked me about the pain, mentioned all the pain* [I] *was suffering. She interrupted she said, “I don’t think* [you] *have that much pain” which is wrong.’—*Interviewee 2.

#### Different forms of subjectivity

Participants suggested that health-care professionals’ subjective opinions held more importance to them than the outcome of scar assessment scales:

*‘What I hear off the doctors and nurses is more important say than the paperwork.’—*Interviewee 4.

This was also reflected by participants’ ability to recall advice and information provided by health-care professionals in greater detail than the content of the scales:

*‘She just said, “Look I really don’t think that’s a good idea because the skin is so fragile at the moment and the scarring is still healing that if we do it now we’re just going to be faced with having to do it again in a couple of months”.’—*Interviewee 5.

The rapport built between patients and their clinical team was found to contribute towards the perception of clinicians’ subjective opinions being more important than scar assessment scales:

*‘I value their opinion a lot … that’s again why it’s nice to see the same people, because they have seen you at your worst.’—*Interviewee 8.

Participants who were further progressed in their burns rehabilitation indicated that they were well informed and confident in self-assessing scarring. They highlighted the importance of their views and how this can be used in conjunction with health-care professionals’ judgements:

*‘I find questionnaires difficult, problematic; I prefer listening to the doctors and what they said and then pushing that up against how I personally feel my body is.’—*Interviewee 9.

### The potential role of objective scar measures

#### Defining an objective measure

Objective scar assessment measures are not currently used in clinical practice, meaning that most participants were reflecting hypothetically on their use. However, two interviewees had first-hand experience of objective scar assessment as prior participants in a research study. They were able to use medical terminology to describe the scar properties being assessed by the tools:

*‘They did three tests, one was to test the elasticity, one was to test the thickness and one was to test the melanin … they showed me the difference between my normal skin and between my scarring and then they put a 3D model together.’—*Interviewee 1.

*‘Using these little gadgets … suck your skin to measure the elasticity in it and things like that, it was like ultrasound … to measure the thickness in scarring.’—*Interviewee 10.

The participants’ ability to recall the scar properties indicates that the tools may contribute towards patient knowledge of scarring, complementing *patient-led care*.

Other participants perceived photography to be an objective measure, indicating that the distinction between objective and subjective is less clear for some assessment strategies.

#### Perceived role of objective measures

Participants who had first-hand experience of objective tools mentioned that the measurements were of interest, but predominantly for the use of health-care professionals:

*‘It’s interesting to see the difference from that to my normal skin … be interesting for the surgeons, so especially with this* [scar] *to measure how deep it’s knitted to me … but that’s for them to look at not me … they need to know so they can treat me.’—*Interviewee 10.

Participants who were asked hypothetical questions surrounding objective scar assessment agreed with this:

*‘It would help assess what further treatment you may or may not need or what different products you could use or medication you could take … to help with the healing process.’—*Interviewee 5.

Interviewee 1 indicated that objective measures may be useful for monitoring scar progression. Note that this is subject to receiving review and comparison with previous results, which was also deemed necessary for scar assessment scales:

*‘If it’s able to tell maybe the difference between last time we went … I think that would be really useful to monitor the progression.’—*Interviewee 1.

#### Objective versus subjective scar assessment

The subjective views of health-care professionals were preferred to objective scar measures by the majority of participants. This related to concepts previously explored such as *continuity in care* and the professional experience of clinicians:

*‘What the doctors are telling me I feel like that’s still very beneficial, and more reassuring to have someone to say that instead of looking at the technology … if you’re sat in front of someone that’s been your doctor for quite a while the trust is there and it’s just a lot more reassuring.’—*Interviewee 1.

The majority of participants viewed objective measures as an additional benefit for clinicians to aid clinical decision making. Interviewee 5 described the holistic process of scar assessment and how objective measures may fit into this:

*‘The doctors and experts for them helping with their own treatment of patients and how to progress things in the future, and on a patient’s level just for their own reassurance … with burns I think it’s a whole multitude of things, it’s the emotional side … it’s the technological side, it’s the research, it’s the questions to see how you’ve progressed.’—*Interviewee 5.

Participants explained the advantages of scar assessment scales, indicating that objective measures may be most beneficial as a supplement rather than replacement for subjective assessments:

*‘The questionnaire was more beneficial because it reads into daily life and how I’m getting on in that respect.’—*Interviewee 1.

Participants explained that objective measures can provide them with further reassurance regarding their scar remodelling, when used in addition to subjective assessments:

*‘It will give me more assurance that what I am doing or the treatment is working.’—*Interviewee 3.

In particular, one participant encountered some difficulties relating to subjective judgements and emphasized how objective scar measures may have been advantageous:

*‘You can see one doctor and then go into another and their views are quite different, and that nearly had a huge impact for me because one doctor wanted to not skin graft me, the other did … I think opinion does come into it and I think it can be very confusing … if everything was factual that would be better.’—*Interviewee 7.

## Discussion

### Key findings and clinical considerations

This study is the first to provide an insight into the patient experience of scar assessment and the use of scar assessment tools during burns rehabilitation. The findings highlight elements of scar assessment clinics that are valued by patients, represented by four subthemes that describe components of patient-centred scar assessment. These are *patient-led care*; *continuity in care*; *learning how to self-manage scarring*; and *psychological assessment*. It became evident that to achieve a patient-centred approach, scar assessment tools should not be detrimental to any of the issues described by these subthemes. Study findings also describe a requirement for a clearer narrative from clinicians regarding the purpose of scar assessment scales and need for a ‘feedback loop’ approach when using the scales, to help patients monitor their scar progression.

A qualitative study by Jones et al. [22] proposed outcome domains important to patients during scar management. Whilst not the main focus of this study and therefore not a detailed component of the analysis, similar outcome domains were discussed by interviewees. For example, *patient-led care* was identified as a strategy for obtaining assessments of outcomes that might influence daily activities, such as movement and function. For outcome domains relating to psychosocial well-being, scar assessment scales were viewed as an opportunity for patients to reflect and communicate their feelings to health-care professionals, thus contributing towards *psychological assessment*. This may be valuable during the early stages of rehabilitation, as interviewees with recently acquired burns described an initial lack of focus on psychological assessment. The importance of *psychological assessment* as a patient-centred component of scar assessment is reflected in the current literature. This includes recognition of emotional functioning as a limiting factor on HRQoL [9] and the importance of support networks, to ease the psychosocial adjustments associated with burns rehabilitation [31].

The use of scar assessment scales as a method of psychological assessment was less greatly emphasized by participants who were more progressed in their burns rehabilitation. This may relate to factors such as patients’ acceptance of scarring, which was expressed by participants who were further along in their rehabilitation. This may lessen the requirement for formal psychological assessments. They may also be less able to recall the level of psychological support that they required and received during the early stages of scar assessment.

*Continuity in care* has been found to provide positive contributions towards care for patients with chronic illnesses [32, 33]; however, it has not been extensively explored in relation to burns rehabilitation. Despite this, some of the related concepts have been identified in previous research. A study by Dahl et al. examined patients’ reflections on care following burn injury [34]. It was noted that having different health-care professionals involved in planning and delivering care contributed towards a ‘lack of support and information’. This example is comparable to those provided by patients in this study and reinforces the value of *continuity in care*.

Patients expressed that receiving advice relating to *learning how to self-manage scarring* was a crucial element of clinic. Litchfield et al. demonstrated that self-management of scarring can allow patients to be involved in clinical decision making, suggesting that it contributes towards *patient-led care* [35]. This highlights the interplay between the subthemes of patient-centred scar assessment, reflecting burns rehabilitation as a holistic process.

Patients expressed uncertainty regarding the purpose of scar assessment scales and the impact that they have on care. This was pertinent to patients who had been attending clinic for longer, who suggested that the scales were of less relevance. As patients accrue experiential knowledge, they may feel more able to drive *patient-led care*, such that they may not deem the scales necessary to convey their views. Patients indicated that their perceptions on scarring and priorities for scar assessment altered over time, which related to their acceptance of scarring. This concept has been recognized in wider research as a ‘response shift’, whereby patients alter their response to a new construct as they become accustomed to it [30, 36].

To obtain the possible patient-centred contributions from scar assessment scales, clinicians could ensure that they communicate the purpose of the scales and potential patient benefits. In addition, patients expressed that receiving feedback and review of completed scales was desirable. This could be achieved in clinic by establishing a ‘feedback loop’ system whereby patients are reminded of previous scores, which are then compared with their most recent scores. These strategies may assist patients in *learning how to self-manage scarring* and support *patient-led care*, as they will acquire a comprehensive view of scar progression, potentially providing a better understanding of the rationale underlying management strategies. Increasing the frequency of scar assessment scale use is likely to be valuable for patients who are earlier in their burns rehabilitation, who may be inexperienced in interpreting scar symptomology and less likely to have established relationships with clinical teams, limiting *continuity in care*.

Health-care professionals’ subjective judgements were recognized as a key element of scar assessment. Therefore, it is important that scar assessment tools are used to supplement rather than replace clinicians’ professional judgements. This is important as some patients identified elements of scarring that are less adequately assessed through clinicians’ judgements alone, for example, sensory changes. Tools such as the BBSIP, which were derived from qualitative work with patients, may allow a more systematic assessment of non-visible scar properties such as pain and itch [14].

As with the scar assessment scales, objective scar measures were viewed as tools to guide health-care professionals, with variation occurring relating to the perceived patient advantage. Many patients deemed the subjective opinions of health-care professionals to be of greater benefit for understanding scar remodelling than objective tools. This coincides with findings by Lee et al. [18] whereby clinicians expressed concerns that objective tools may overshadow the value of health-care professionals’ subjective judgements.

Although objective tools are not established in clinical practice, patients were able to understand the differences in the aspects of scarring assessed by objective tools compared with subjective measures. Patients provided example scenarios where objective measures would have been advantageous to resolve discrepancies amongst subjective measures, where the judgements of clinicians and patients may be in opposition. Clinicians could support *patient-led care* by identifying scar properties that are central to patients’ healing and use the objective tools to provide detailed measurements of this. For patients who are confident in leading scar assessment, it may be appropriate to incorporate their views when selecting assessment tools, to focus scar assessment on patient-centred priorities.

### Future research

The exploratory nature of this study has indicated areas for further research, to obtain a more comprehensive understanding of scar assessment as an integral part of burns rehabilitation. The variation in viewpoints between patients with older and more recently acquired scarring became a key associative analysis. Further exploration into how patients’ perceptions alter as they gain experiential knowledge may provide a greater understanding of these observed differences. Research into health-care professionals’ experiences would provide a deeper insight into the interactions between clinicians and patients and how scar assessment tools may influence this. In addition, research investigating how scar assessment scales may facilitate assessments of psychosocial symptoms would provide insight into the potential role of scales in monitoring patients’ psychological rehabilitation. As the use of objective measures is mainly within the context of research, conclusions made regarding their use are tentative. Further research is desirable, focusing on the patient-centred impact of objective tools when used in clinical practice.

### Strengths and limitations

This is the first primary study to consider patients’ views on scar assessment. It has provided a novel insight into core components that enable a patient-centred approach and explored how assessment tools may contribute towards this. The research has formed recommendations that may help to optimize the use of scar assessment scales and has demonstrated the potential role of objective measures. However, as noted earlier, due to the hypothetical nature of most of the discussions regarding objective tools, further research is warranted. For two of the participants, involvement in research relating to objective measures may have influenced their understanding and views of scar assessment. In addition, the interviewer’s trainee medical background may have influenced the way in which they described the objective measures. Strategies were used to acknowledge this, including completion of a reflexive diary and discussion of alternate interpretations with experienced qualitative researchers.

The study sample was diverse with regards to burn characteristics and demographic measures such as age. In particular, this study has provided a unique insight into the value of scar assessment for patients at various stages of burns rehabilitation. It is important to note the gender imbalance within the sample. However, associative analyses demonstrated commonality in thematic content between male and female participants. Participation was limited to patients from one clinical trust, meaning their experiences may differ from the wider patient population and further research is required to investigate this. However, as participants were not all under the care of the same clinical teams, they will have experienced variation in the way in which clinicians conduct scar assessment.

## Conclusions

This exploratory qualitative study identified four key subthemes that contribute towards conducting scar assessment in a patient-centred manner. Scar assessment scales can provide patients with opportunity for psychological reflection and objective tools may aid patients’ understanding of scar properties. However, the subjective judgements of health-care professionals were regarded as more important and misconceptions were noted relating to the purpose of assessment tools. To optimize the use of scar assessment scales, clinicians should clearly communicate the purpose of the scales. Establishing a ‘feedback loop’ system whereby completed scales are reviewed may be beneficial to provide a holistic view of scar progression and assist inexperienced patients in their understanding of scar symptomology. Further research into health-care professionals’ views on scar assessment tools is desirable, to obtain a complete understanding of their role during burns rehabilitation.

## Abbreviations

BBSIP: Brisbane Burn Scar Impact Profile; HRQoL: health-related quality of life; POSAS: Patient and Observer Scar Rating Scale; PPI: patient and public involvement; VSS: Vancouver Scar Scale

## Authors’ contributions

KP, JM and NM jointly conceived the study. KP designed the study with JM’s support, obtained relevant ethical approvals, conducted patient interviews, data analysis and wrote the initial draft of the manuscript. JM supported KP in study design, ethical approvals, data analysis and made substantial amendments to the manuscript. NM and LN assisted in study design, patient and public involvement and review of the manuscript.

## Supplementary Material

Supplementary_file_1-_Interview_topic_guide_tkab005

## Data Availability

Not applicable.
