# Supplementary material for: Patient experience of scar assessment and the use of scar assessment tools during burns rehabilitation: a qualitative study
Source: Burns Trauma. 2021 Jun 1;9:tkab005. doi: 10.1093/burnst/tkab005 (PMC8240530; doi:10.1093/burnst/tkab005)
Supplement: Supplementary_file_1-_Interview_topic_guide_tkab005 [file supplementary_file_1-_interview_topic_guide_tkab005.docx]

**Supplementary file 1: Interview topic guide**

**Introduction**

- Introduce researcher and explain background
  - Hello my name is [name], I am a medical student from the University of Birmingham
- Thank participant for attending the interview and participating in the study
- Explain the purpose and rationale for the study
  - I am interested in learning what it’s like for people when they come to clinic and have their burn scars looked at and I want to find out from a patient point of view how useful the current techniques are to assess scarring. This project is being carried out as part of my intercalated degree in public health.
- Interview structure
  - During the interview I will talk to you about how you acquired your burn injury and your experience of attending clinic to have your scars assessed. I will also be asking questions about the different methods used to assessed scars. The interview is expected to last between 30-60 minutes, however you can pause or end the interview at any point if you wish.
- Data collection
  - This interview will be digitally recorded and written up by a 3^rd^ party transcriber who has signed a confidentiality agreement. I will also ask you to fill out a short questionnaire about yourself before we begin.
- Confidentiality
  - Anything you say during this interview will be kept strictly confidential and you will not be identified on the written record of the interview.
- Questions
  - Do you have any questions or concerns before we begin?
- Ask the participant to complete the consent form or verbally read out the statements from the consent form (for telephone interviews)
- Ask participant to complete the background questionnaire or verbally work through the questionnaires (for telephone interviews)
- Check that the participant is ok to start the interview

**Ice breaker and burn injury**

- If you’re happy to talk about it would you mind tell me a bit about your burn injury?
  - Whereabouts on your body you were injured?
  - How long ago it happened?
  - What treatments have you had?

**Experience of attending outpatient scar assessment clinic**

- How often have you been attending clinic for scar assessment?
- Would you be able to talk me through what happens when you go to clinic?
- Who do you see?
- What do they do when they look at your scarring?
- Can you describe how they assess how things are progressing?
- Did they talk to you about some of the assessments they were doing?

**Scar assessment scales**

**Check understanding and awareness**

- Have you noticed that the [use patient terminology: doctors/nurse/physios etc] have used any questionnaire-style forms called scar assessment scales to assess your scarring?
- How often were they used?
- Have you filled them in yourself?

**The role of scar assessment scales**

- Why do you think scar assessment scales were used by [healthcare professional]?
- Do you know what types of things are assessed on the scar assessment scales, do you think any of these are more important than others?
- What role do you see scar assessment scales playing for you personally?
- How do you feel when asked to complete a scar assessment scale?
- How do you feel when you are filling out the scar assessment scales?
- Do you use the scales to monitor how your scars are healing?
- How are the scales used once you have completed them? Do you ever look back on them?

**Scar assessment scales vs healthcare professionals’ subjective judgements**

- What sort of feedback have you had from [healthcare professional] about how your healing is progressing?
- How do you know that your scars are getting better/worse?
- How does the opinion of [healthcare professional] compare to the scar assessment scales? Do you value them equally?
- Does the feedback of [healthcare professional] agree with how you feel about how your scars and healing?

**Objective scar measures**

- Explain what objective scar measures are, providing examples of equipment and a brief overview of how this works (adapt to perceived understanding of each patient)
- Have you ever had any technology used to assess your scarring?
- Do you think technology is needed to assess scars?
- How would the use of technology compare to what [healthcare professional/ scar assessment scales] says about your scarring?
- Would having accurate recording of scar properties [give example e.g. an accurate recording of scar thickness] be useful for you?

**Important factors relating to scarring**

- What sort of things do you want assessing when you come to clinic to see [healthcare professional]?
- Do you think that these are currently being assessed thoroughly in clinic? (ask how this is being conducted)
- Is there anything that you feel is not being assessed during your clinic appointments?
- What needs to change about your scarring for you to know that it is healing?
- What part of the scarring process has had the biggest influence on your daily life? Has this been assessed during clinic? (ask how this is being conducted)

**End of interview**

- Ask participant if they would like to add anything else
- Thank participant
- Write up any relevant field notes
